# Supplementary material for: Neural correlates of hierarchical predictive processes in autistic adults
Source: Nat Commun. 2023 Jun 19;14:3640. doi: 10.1038/s41467-023-38580-9 (PMC10279690; doi:10.1038/s41467-023-38580-9)
Supplement: Supplementary file 1 — Supplementary Information [file 41467_2023_38580_MOESM1_ESM.pdf]

Inventory of Supporting Information

Supplementary Figure S1: Neural correlates of prior precisions (p. 1)

Supplementary Figure S2: Contrast Ambiguous rotation vs. baseline masked with the MT/V5 mask (p. 2)

Supplementary Table S1: Model parameters (p. 3)

Supplementary Table S2: Positive effect of the prior mean on brain activity (pp. 4-6)

Supplementary Table S3: Positive effect of the prior precision on brain activity (p. 7)

Supplementary Table S4: Positive effect of the prediction errors on brain activity (pp. 8-9)

Supplementary Table S5: Correlations between the questionnaire scores and the contrast estimates in the clusters showing group differences (p. 10)

Supplementary Note S1: Full mathematical model description (pp. 11-16)

Supplementary Note S2: Model and parameter recoveries (p. 17)

Supplementary Note S3: Activation by ambiguous trials in MT/V5 (p. 18)

Supplementary References (p. 19)

Supplementary information

Supplementary Figure S1: Neural correlates of prior precisions

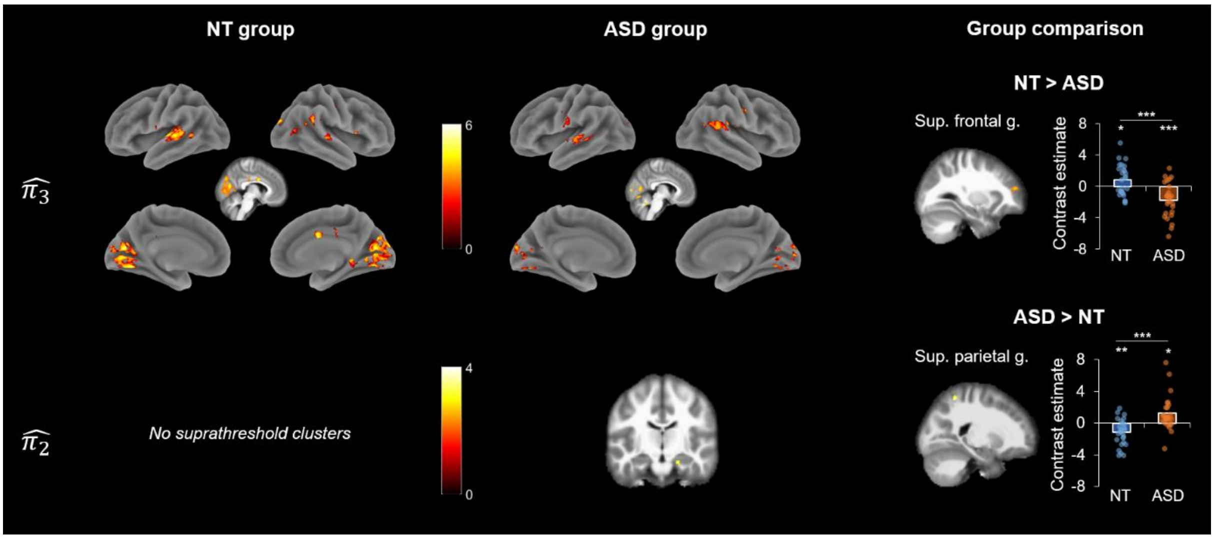

Top row: Positive effect of the 3rd level prior precision $\widehat{\pi_3}$ in the NT and ASD groups, and group difference in the left superior frontal gyrus. Significance level was set at $p < .001$ at voxel level and $p < .05$ at cluster level. Sup. frontal g. histogram: : one-sample t-test in NT: $p < .046$, in ASD: $p < .001$; two-sample t-test: $p < .001$.

Bottom row: Positive effect of the 2nd level prior mean $\widehat{\pi_2}$ in the NT group (no suprathreshold cluster with a significance level of $p < .001$ at voxel level) and in the ASD group (the significance level was set at $p < .001$ at voxel level but at $p < .08$ at cluster level here, the cluster was not significant for $p < .05$ at cluster level). Right: group difference in the right superior parietal lobule: one-sample t-test in NT: $p = .001$, in ASD: $p = .011$; two-sample t-test: $p < .001$.

In the histograms, the significance level of the two-sample t-tests and one-sample t-tests (two-sided) are indicated as follows: * $p < .05$, ** $p < .01$, *** $p < .001$. NT group: n = 26, ASD group : n = 25.

Supplementary Figure S2: Contrast Ambiguous rotation vs. baseline masked with the MT/V5 mask

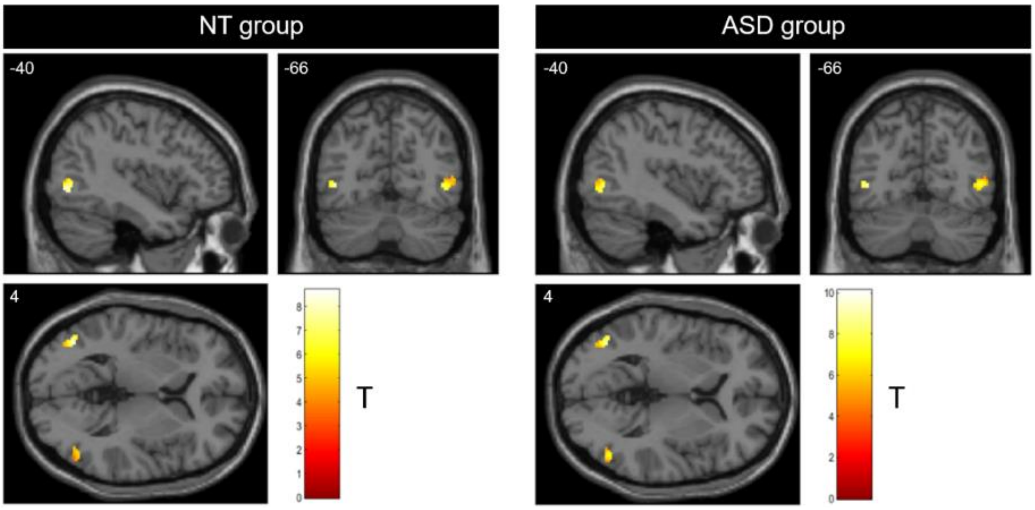

The contrast *Ambiguous rotation* vs. *baseline* were masked with this MT/V5 mask, and voxel-based level thresholding was set at $p < .001$, while cluster-level extent thresholding was set at $p < .05$. NT group: n = 26, ASD group : n = 25. Details are given in the Supplementary note 3.

Supplementary Table S1: Model parameters

Model parameters		Prior	Posterior	
			NT group	ASD group
Perceptual model				
π_a	Associative learning precision	0.50 (1.00)	1.37 (0.28)	1.36 (0.28)
π_s	Sensory memory precision	0.50 (1.00)	0.25 (0.59)	0.36 (0.30)
π_p	Priming precision	0.50 (1.00)	0.10 (0.13)	0.09 (0.10)
π_{dis}	Disambiguation precision	1.50 (0.00)	1.50 (0.00)	1.50 (0.00)
Contingency model				
ω_2	Second-level learning rate	-1.28 (1.00)	-0.72 (0.86)	-0.89 (1.28)
ω_3	Third-level learning rate	-6.14 (1.00)	-6.23 (0.09)	-6.25 (0.11)
κ_2	Coupling strength between 3rd and 2nd levels	1.00 (0.00)	1.00 (0.00)	1.00 (0.00)
μ_2	Initial mean of the 2nd level	0.00 (0.00)	0.00 (0.00)	0.00 (0.00)
μ_3	Initial mean of the 3rd level	1.00 (0.00)	1.00 (0.00)	1.00 (0.00)
σ_2^0	Initial variance of the 2nd level	4.64 (1.00)	4.58 (0.01)	4.13 (0.01)
σ_3^0	Initial variance of the 3rd level	4.00 (1.00)	3.77 (0.01)	3.13 (0.02)
Response mapping				
ζ	Inverse decision parameter	1.00 (0.00)	1.00 (0.00)	1.00 (0.00)

Mean and variance of Gaussian priors used to model the data, and their posterior estimates using Bayesian Model Averaging. The prior parameters are the same as in [1]. Values are presented as Mean (variance) for the prior values and Mean (standard deviation) for the posterior values.

Supplementary Table S2: Positive effect of the prior mean on brain activity

**S2.1. Third-level prior mean $\lvert\hat{\mu}_3\rvert$**							
**Region**		**Size**	**T**	***p* (cluster)**	**MNI coordinates**		
					**x**	**y**	**z**
**NT group**							
**Cerebellum**	**R**	112	6.37	< .001	30	-58	-26
**SMA**	**L, R**	647	5.95	< .001	6	24	50
**Middle/sup. temporal g. / TPJ**	**R**	372	5.37	< .001	66	-40	8
**Insula**	**L**	81	5.33	.002	-34	16	-4
**Precentral g.**	**L**	109	5.13	.001	-36	2	32
**Middle frontal g.**	**R**	227	5.12	< .001	42	6	36
Cerebellum	L	33	4.81	.033	6	-74	-24
**Inf. parietal lobe**	**L**	122	4.54	< .001	-44	-42	38
**Inf. parietal lobe**	**R**	303	4.48	< .001	50	-46	52
Middle (/post.) cingulate	R	71	4.23	.003	6	-20	38
Postcentral	L	60	4.14	.006	-46	-26	60
Inf. temporal sulcus (post.)	L	40	4.87	.021	-56	-46	-12
Middle temporal g.	R	32	5.20	.035	40	-62	12
Sup. temporal g. (post.)	L	35	3.91	.029	-64	-44	16
Inf. frontal g.	R	37	3.70	.025	56	10	0
**ASD group**							
**Sup. temporal g. (post)**	**R**	239	5.08	< .001	64	-38	6
**SMA**	**L, R**	257	4.88	< .001	6	16	56
**Inf. frontal g.**	**R**	129	4.85	< .001	46	26	18
Middle cingulate	L, R	49	4.67	.012	2	-14	40
**Post. (middle) cingulate**	**L, R**	175	4.53	< .001	0	-26	32
**Inf. frontal g.**	**L**	170	4.53	< .001	-46	8	20
**Middle temporal g. (post)**	**L**	147	4.44	< .001	-64	-30	2
Middle cingulate	R	37	4.43	.025	6	22	38
Sup. temporal g. (post)	R	34	4.06	.031	62	-16	-8
Insula	R	57	4.04	.007	34	26	-4
Angular g.	R	40	3.87	.021	32	-62	38
**ASD > NT**							
Orbitofrontal cortex	L	39	4.54	.022	-30	34	-16
Post. cingulate	L	54	4.42	.009	6	-50	16
Post. cingulate	R	33	3.69	.033	8	-50	32
Sup. temporal g. (post.)	R	30	4.05	.041	40	-32	16

**S2.2. Second-level prior mean $|\hat{\mu}_2|$**							
**Region**		**Size**	**T**	***p* (cluster)**	**MNI coordinates**		
					**x**	**y**	**z**
**NT group**							
**Post./middle/ant. Cingulate / SMA / Medial frontal sup. / orbital / Precuneus / Retrosplenial / Cuneus / Calcarine / Lingual / Sup. occipital / Hippocampus / Parahippocampal / Amygdala / Paracentral lobule Ventral striatum / Caudate / Cerebellum**	**L, R**	11845	8.27	< .001	14	-50	4
**Sup. frontal g.**	**L**	201	5.65	< .001	-22	36	46
Inf. frontal g. orbital / OFC	L	56	4.35	.010	-26	30	-12
**Precentral g.**	**R**	491	5.59	< .001	44	-16	60
**Postcentral g.**	**L**	126	4.69	< .001	-46	-12	58
**Sup. temporal g. / Rolandic / Insula**	**L**	1507	6.54	< .001	-44	-20	2
**Sup. temporal g. / Rolandic / Insula**	**R**	1094	5.77	< .001	54	-4	-12
**Middle temporal pole**	**R**	127	6.10	< .001	44	10	-32
Sup. temporal pole / amygdala	R	69	5.66	.005	32	4	-20
Sup. temporal g. / Rolandic	R	78	5.25	.003	44	-34	18
**Middle temporal g. (post.) / pSTS**	**L**	358	5.54	< .001	-40	-60	18
Middle/Inf. temporal g. (ant.)	L	65	5.13	.006	-44	6	-30
Insula	R	61	4.61	.007	40	6	12
**Lingual / Cerebellum**	**L**	181	4.21	< .001	-22	-56	-16
**ASD group**							
**Lingual / Calcarine / Precuneus / Retrosplenial / Cuneus / Sup. Occipital / Post. Cingulate / Hippocampus / Parahippocampal / Cerebellum**	**L, R**	6421	8.53	< .001	22	-46	10
**Medial orbital frontal / ACC pre**	**L, R**	981	5.72	< .001	2	22	-4
Inf. Frontal g. orbital / OFC	L	35	4.26	.034	-28	38	-6
**Precentral**	**R**	133	5.09	< .001	42	-12	50
**Middle cingulate / Precuneus**	**L, R**	1024	5.81	< .001	16	-38	52
**Middle/Sup. Temporal g. and pole**	**L**	370	6.48	< .001	-60	-2	-12
**Middle temporal g. (post.)**	**L**	156	5.29	< .001	-50	-68	12
Middle temporal g.	R	43	4.07	.021	54	-64	4
Middle / sup. temporal pole	R	30	4.25	.047	46	16	-24
**Rolandic / Sup. temporal g.**	**L**	151	4.49	< .001	-50	4	4
**Ventral striatum**	**L, R**						
**Insula / Rolandic**	**R**	1279	5.56	< .001	60	0	-10
Insula / Rolandic	L	35	4.38	.034	-34	6	10
Sup. parietal	L	58	4.25	.009	-26	-38	58
Angular g.	L	39	3.78	.026	-48	-66	26
Sup. occipital	L	39	4.10	.026	-20	-88	22
Cuneus / Sup. occipital	L	58	4.08	.009	-12	-88	14
**ASD > NT**							
Retrosplenial complex	R	32	5.66	.041	20	-46	10

**S2.3. Inferred conditional probability of a clockwise rotation given the tone μ_a**							
**Region**		**Size**	**T**	***p* (cluster)**	**MNI coordinates**		
					**x**	**y**	**z**
**NT group**							
**Lingual / Fusiform / Middle occipital**	**R**	672	5.75	< .001	28	-70	-8
Postcentral	L	29	4.01	.039	-42	-24	54
**ASD group**							
**Lingual / Fusiform / Inf. occipital**	**R**	99	4.67	.001	22	-80	-8
Postcentral	L	47	3.85	.011	-36	-24	50

In the three tables above, significance level was set at $p < .001$ at voxel level and $p < .05$ at cluster level. Regions remaining significant after FWE correction at $p < .05$ at cluster level appear in bold. Within group, contrasts across participants were compared to zero using one-sample Student's t-test. NT and ASD groups were compared using two-sample Student’s t-tests.

L: Left, R: Right, g.: gyrus, s.: sulcus ant.: anterior, post.: posterior, inf.: inferior, sup.: superior, SMA: Supplementary Motor Area, ACC: Anterior Cingulate Cortex, OFC: Orbitofrontal cortex, TPJ: Temporo-Parietal Junction.

Supplementary Table S3: Positive effect of the prior precision on brain activity

**S3.1. Third-level prior precision $\widehat{\pi_3}$**							
**Region**		**Size**	**T**	***p* (cluster)**	**MNI coordinates x**	**y**	**z**
**NT group**							
**Calcarine / Lingual / Cuneus**	**L, R**	3539	6.52	< .001	-2	-70	12
Lingual	R	58	4.93	.009	14	-42	-4
**TPJ**	**R**	164	4.55	< .001	54	-44	24
**Sup. temporal g.**	**L**	930	5.63	< .001	-60	-36	8
**Sup. temporal g.**	**R**	98	4.29	.001	64	-22	-2
**Middle temporal g.**	**R**	101	4.73	.001	42	-64	10
**Middle cingulate**	**L, R**	151	6.06	< .001	4	0	30
Middle cingulate	R	39	4.13	.027	8	-24	34
**Inf. frontal g. / Insula**	**R**	102	4.79	.001	44	10	0
Insula	L	58	4.56	.009	-40	14	4
SMA	L, R	30	3.72	.048	0	8	66
**ASD group**							
**Sup. temporal / TPJ**	**R**	440	5.75	< .001	52	-40	20
**Middle temporal g.**	**L**	93	4.40	.002	-60	-24	0
Middle temporal g.	L	80	4.47	.003	-50	-34	0
Temporal pole (sup.)	L	73	4.87	.004	-38	18	-18
**Calcarine / Cuneus**	**L, R**	294	5.63	< .001	-12	-90	16
**Calcarine**	**L, R**	177	4.28	< .001	8	-70	8
Cuneus	R	77	4.11	.003	14	-92	26
**Cerebellum / Lingual**	**L, R**	753	5.55	< .001	-8	-66	-10
Cerebellum	R	36	4.44	.033	6	-52	-28
**Postcentral g.**	**L**	106	4.15	.001	-58	-10	26
**Central sulcus**	**R**	128	4.42	< .001	46	-12	32
Pallidum	R	70	5.21	.005	16	4	-4
**NT > ASD**							
Sup. frontal g.	L	35	4.44	.035	-30	52	18

**S3.2. Second-level prior precision $\widehat{\pi_2}$**							
**Region**		**Size**	**T**	***p* (cluster)**	**MNI coordinates x**	**y**	**z**
**ASD group**							
Parahippocampal cortex #	R	21	3.96	.080	20	-16	-22
**ASD > NT**							
Sup. parietal	R	26	4.55	.054	22	-50	62

In the two tables above, significance level was set at $p < .001$ at voxel level and $p < .05$ at cluster level, except for # where it was set at $p < .08$ at cluster level. Within group, contrasts across participants were compared to zero using one-sample Student's t-test. NT and ASD groups were compared using two-sample Student's t-tests. Regions remaining significant after FWE correction at $p < .05$ at cluster level appear in bold. L: Left, R: Right, g.: gyrus, sup.: superior, SMA: Supplementary Motor Area, TPJ: Temporo-Parietal Junction.

Supplementary Table S4: Positive effect of the prediction errors on brain activity

**S4.1. Third-level precision-weighted prediction error $\lvert\varepsilon_3\rvert$**							
**Region**		**Size**	**T**	***p* (cluster)**	**MNI coordinates**		
					**x**	**y**	**z**
**NT group**							
Middle cingulate	L, R	35	5.17	.026	-2	-30	38
Post. cingulate	L, R	33	4.50	.030	2	-42	20
**ASD group**							
Middle temporal g. (post)	L	38	5.42	.021	-62	-8	-8
**Middle temporal g. (post)**	**L**	192	4.81	< .001	-62	-48	-8
Middle temporal g. (post)	R	47	4.16	.012	54	-42	0
Angular g.	L	63	4.54	.004	-44	-64	48
Angular g.	R	34	3.84	.028	44	-62	44
Sup. Frontal g.	L	68	4.35	.003	-12	50	38

**S4.2. Second-level precision-weighted prediction error $\lvert\varepsilon_2\rvert$**							
**Region**		**Size**	**T**	***p* (cluster)**	**MNI coordinates**		
					**x**	**y**	**z**
**NT group**							
**SMA / Sup. medial frontal / Middle cingulate**	**L, R**	1725	7.35	< .001	28	10	66
**Middle frontal g.**	**R**	576	6.13	< .001	40	26	42
**Middle frontal g.**	**L**	160	5.41	< .001	-42	26	30
**Sup. frontal g.**	**L**	347	5.12	< .001	-22	-8	58
**Inf. parietal / Angular g.**	**R**	576	5.63	< .001	40	-60	42
**Inf. parietal**	**L**	256	4.60	< .001	-46	-48	46
**Precuneus**	**L, R**	361	5.81	< .001	4	-66	46
Middle temporal g.	R	58	4.95	.007	66	-28	-6
Insula	L	30	4.16	.040	-32	24	-2
Caudate nucleus	L	27	4.81	.050	-18	2	18
**ASD group**							
**SMA**	**L**	194	4.40	< .001	-4	20	48
Sup. frontal g. / SMA	R	29	3.85	.043	20	10	68
**Middle/sup. frontal g.**	**R**	128	4.52	< .001	32	8	56
Middle/sup. frontal g.	L	54	3.86	.009	-24	10	48
Sup. frontal g.	L	51	4.07	.010	-16	26	52
Middle frontal g.	L	38	4.40	.023	-40	14	52
Inf. frontal g.	L	49	4.87	.012	-30	20	30
**Inf. parietal / Angular g.**	**L**	375	5.12	< .001	-50	-58	44
**Inf. parietal / Angular g.**	**R**	276	4.81	< .001	50	-54	44
**Insula**	**L**	85	4.72	.002	-38	14	-4
Caudate nucleus	L	57	4.91	.007	-14	12	8
**ASD > NT**							
**ACC**	**L**	95	4.20	.001	-14	46	6
Putamen	L	26	4.15	.054	-30	2	0

**S4.3. Absolute perceptual prediction error $|\delta_q|$**							
**Region**		**Size**	**T**	***p* (cluster)**	**MNI coordinates**		
					**x**	**y**	**z**
**NT group**							
**Precentral g. / Inf. frontal g.**	**L**	2104	8.08	< .001	-26	-2	50
**Precentral g. / Inf. frontal g.**	**R**	2387	7.84	< .001	42	6	26
**SMA**	**L, R**	1043	6.76	< .001	-6	14	50
ACC sup.	L, R	38	4.61	.028	-2	10	28
**Inf./sup. parietal**	**L**	2587	6.58	< .001	-32	-42	42
**Supramarginal / Inf. parietal**	**R**	2414	6.53	< .001	30	-72	36
Inf. parietal	L	56	4.84	.010	-40	-46	54
**Insula**	**L**	228	6.73	< .001	-28	26	2
**Post. inf./middle temporal g.**	**L**	1280	6.87	< .001	-46	-64	-14
**Post. inf./middle temporal g.**	**R**	1131	8.11	< .001	48	-60	-16
Middle temporal g.	R	62	4.58	.007	44	-60	12
Sup. temporal s.	R	43	4.47	.021	52	-28	-8
Middle occipital	R	43	4.31	.021	38	-80	12
**ASD group**							
**Precentral g. / Inf. frontal g.**	**L**	1109	7.81	< .001	-42	6	32
**Inf. Frontal g. / Insula**	**R**	2515	8.23	< .001	34	26	-2
**Inf. Frontal g. / Insula**	**L**	482	6.22	< .001	-32	24	-2
Middle/inf. frontal g.	L	63	4.11	.007	-40	46	18
**Sup./middle frontal g.**	**L**	305	5.47	< .001	-24	0	50
**SMA**	**L, R**	1304	7.82	< .001	8	16	48
**Inf./sup. parietal / Precuneus**	**L, R**	4410	7.21	< .001	14	-70	52
**Supramarginal / Inf. parietal g.**	**L**	298	5.17	< .001	-44	-42	52
**Inf./middle temporal g. (post.)**	**L**	478	4.87	< .001	-52	-60	-2
**Inf./middle temporal g. (post.)**	**R**	848	5.59	< .001	56	-58	-4
Middle temporal g.	L	75	4.40	.004	-58	-48	8

In the three tables above, significance level was set at $p < .001$ at voxel level and $p < .05$ at cluster level. Regions remaining significant after FWE correction at $p < .05$ at cluster level appear in bold. Within group, contrasts across participants were compared to zero using one-sample Student's t-test. NT and ASD groups were compared using two-sample Student's t-tests.

L: Left, R: Right, g.: gyrus, s.: sulcus ant.: anterior, post.: posterior, inf.: inferior, sup.: superior, SMA: Supplementary Motor Area, ACC: Anterior Cingulate Cortex.

Supplementary Table S5: Correlations between the questionnaire scores and the contrast estimates in the clusters showing group differences (r values)

		**AQ**	**IU**	**GSQ**
$\lvert\hat{\mu}_3\rvert$	Orbitofrontal cortex (L)	ns	0.33 * ($p_{FDR\text{-}corr}$ = .030)	ns
	Posterior cingulate cortex (L, R)	0.30 * ($p_{FDR\text{-}corr}$ = .044)	0.35 * ($p_{FDR\text{-}corr}$ = .021)	ns
	Superior temporal sulcus (post) (R)	0.44 ** ($p_{FDR\text{-}corr}$ = .004)	0.41 ** ($p_{FDR\text{-}corr}$ = .008)	0.33 * ($p_{FDR\text{-}corr}$ = .030)
$\lvert\hat{\mu}_2\rvert$	Retrosplenial cortex (R)	0.46 ** ($p_{FDR\text{-}corr}$ = .004)	0.40 *** ($p_{FDR\text{-}corr}$ = .009)	ns
$\hat{\pi}_3$	Superior frontal gyrus (L)	ns	ns	ns
$\hat{\pi}_2$	Superior parietal lobe (R)	0.51 ** ($p_{FDR\text{-}corr}$ = .001)	0.39 * ($p_{FDR\text{-}corr}$ = .011)	0.42 ** ($p_{FDR\text{-}corr}$ = .007)
$\lvert\varepsilon_2\rvert$	Anterior cingulate cortex (L)	0.45 ** ($p_{FDR\text{-}corr}$ = .004)	0.30 * ($p_{FDR\text{-}corr}$ = .044)	ns
	Putamen (L)	0.40 ** ($p_{FDR\text{-}corr}$ = .009)	0.42 ** ($p_{FDR\text{-}corr}$ = .007)	0.33 * ($p_{FDR\text{-}corr}$ = .030)

Correlations were assessed using Pearson's correlation test, and corrected for multiple comparisons using False Discovery Rate (FDR) correction. * $p < .05$, ** $p < .01$ for p-values adjusted after FDR correction. ns: non-significant.

AQ: Autism-spectrum Quotient, IU: Intolerance of Uncertainty scale, GSQ: Glasgow Sensory Questionnaire.

Supplementary Note 1: Full mathematical model description

This section is similar to the one described in the article by Weilnhammer and colleagues, 2018[1]. The priors of the parameters are described in Table S1 and are similar to the ones of Weilnhammer and colleagues, 2018[1].

Perceptual model

At each time point *t*, the two alternative visual percepts are predicted on the basis of a posterior probability distribution over θ:

$$\theta = \begin{cases} > 0.5 : \ CW\ tilt \\ < 0.5 \ : CCW\ tilt \end{cases} \tag{1}$$

Participants responded with button presses indicating the current visual percept as follows:

$$y_{perception}(t) = \begin{cases} 1 : CW\ tilt \\ 0 : CCW\ tilt \end{cases} \tag{2}$$

Based on previous work[1,2], we formalized a number of prior distributions that could influence on participants' perception, considering separate contributions of priming, sensory memory, and associative learning. The latter was driven by the co-occurrence of the direction of tilt (see above) and the pitch of the preceding tone, which was defined as follows:

$$\beta(t) = \begin{cases} 1 : high\, pitch \\ 0 : low\ pitch \end{cases} \tag{3}$$

To map the dynamic inference on the contingency between tones β and perceived direction of tilt y, we constructed a three-level hierarchical Gaussian filter (Mathys et al. (2014b), see below for details), which received the conjunction of tone and posterior probability of tilt direction as input. From here, we extracted first level prediction $\hat{\mu}_1(t)$, which represents the inferred contingency over tones and rotations. This was transformed into the conditional probability of CW tilt given the tone as follows:

$$\mu_a(t) = \begin{cases} \hat{\mu}_1(t) : for\ \beta(t) = 0 \\ 1 - \hat{\mu}_1(t) : for\ \beta(t) = 1 \end{cases} \tag{4}$$

This defines the mean of the prior distribution "associative learning" (associative learning ~ N(μ_a, π_a^{-1})), while π_a represents its precision. Please note that the conditional probability of CCW tilt is given by $1 - \mu_a$.

Likewise, the mean of the prior distribution "priming" (priming ~ N(μ_p, π_p^{-1})) in trial *t* was defined by the visual percept in the preceding trial:

$$\mu_a(t) = y_{perception}(t-1) \tag{5}$$

The mean of the prior distribution "sensory memory" (sensory memory ~ N(μ_s, π_s^{-1})) in trial *t* was defined by the visual percept in the preceding ambiguous trial t_a:

$$\mu_s(t) = y_{perception}(t_a) \tag{6}$$

In addition to these prior distributions, we defined the disambiguation (i.e., the presence of motion streaks along the trajectory of tilt) by means of the likelihood weight "disambiguation" (disambiguation ~ N($\mu_{dis}, \pi_{dis}^{-1}$)) in trial *t*:

$$\mu_{dis}(t) = \begin{cases} 1 : CW\ (disambiguation) \\ 0.5 : CW/CCW\ (ambiguous) \\ 0 : CCW\ (disambiguation) \end{cases} \tag{7}$$

To predict the perceptual outcomes, we derived the posterior distribution with respect to CW or CCW tilt from the model. This distribution results from a weighting of a bimodal likelihood distribution by a combination of prior distributions such as "associative learning", "priming", "sensory memory", as well as the likelihood weight "disambiguation".

For a specific combination of these prior distributions, a joint prior distribution with mean μ_m and precision π_m can be calculated by adding up the means of influencing factors relative to their respective precision:

$$\mu_m(t) = \frac{\pi_a\mu_a(t) + \pi_p\mu_p(t) + \pi_s\mu_s(t)}{\pi_m} \tag{8}$$

$$\pi_m = \pi_a + \pi_p + \pi_s \tag{9}$$

This joint prior distribution (described by μ_m and π_m) as well as the disambiguation (defined by μ_{dis} and π_{dis}) is used to adjust the density ratio of the posterior for the two peak locations $\theta_0 = 0$ and $\theta_1 = 1$:

$$r(t) = \frac{P(\theta_1(t))}{P(\theta_0(t))} \tag{10}$$

$$= \exp\left(\frac{(\theta_1 - \frac{\pi_m\mu_m(t) + \pi_{dis}\mu_{dis}(t)}{\pi_m + \pi_{dis}})^2 - (\theta_0 - \frac{\pi_m\mu_m(t) + \pi_{dis}\mu_{dis}(t)}{\pi_m + \pi_{dis}})^2}{2 * (\pi_m + \pi_{dis})^{-2}}\right)$$

$$P(\theta_1) = \frac{1}{r(t) + 1} \tag{11}$$

$P(\theta_1)$ denotes the posterior probability of CW tilt. Therefore, 1 - $P(\theta_1)$ represents the posterior probability of CCW tilt.

The model prediction $\hat{y}_{perception}$ on the participants percept is given by applying a unit sigmoid function with inverse decision temperature $\zeta = 1$ to $P(\theta_1)$:

$$\hat{y}_{perception} = \frac{P(\theta_1)^\zeta}{P(\theta_1)^\zeta + (1 - P(\theta_1))^\zeta} \tag{12}$$

From here, we extracted a “perceptual prediction error”, which was given by:

$$\delta_q = P(\theta_1) - y_{perception} \tag{13}$$

In addition, we defined a “choice prediction error”, which was obtained by subtracting the inferred conditional probability of CW tilt given the tone (i.e., μ_a) from the actual perceptual outcome $y_{perception}$:

$$\varepsilon_{choice} = \mu_a - y_{perception} \tag{14}$$

Contingency model

To extract the inferred trial-by-trial prediction $\hat{\mu}_1(t)$, we used a version of the three-level hierarchical Gaussian filter[3]. The input to the HGF modeling the inferred contingency between auditory and visual stimuli was defined by the following:

$$Input(t) = |P(\theta_1(t)) - \beta(t)| \tag{15}$$

Updates in the inferred contingency are smaller in ambiguous cases and the HGF implemented here specifically takes differences in perceptual certainty between ambiguous and unambiguous trials into account.

Likewise, the participants' prediction was defined as follows:

$$y_{prediction}(t) = \begin{cases} |1 - \beta(t)|: CW\ tilt \\ |0 - \beta(t)|: CCW\ tilt \end{cases} \tag{16}$$

The posterior of the first level $\mu_1(t)$ is set to be equal to $Input(t)$:

$$\mu_1(t) = Input(t) \tag{17}$$

The second-level prediction of the HGF models the tendency of the first level toward $\mu_1(t) = 1$ and is given by the following:

$$\mu_2(t) = \hat{\mu}_2(t) + \frac{1}{\pi_2(t)} * \delta_1(t) \tag{18}$$

$$\hat{\mu}_2(t) = \mu_2(t-1) \tag{19}$$

Please note that we refer to the strength of the second-level prediction $|\hat{\mu}_2(t)|$ as "mid-level" prediction. The precision of the second-level prediction evolves according to the following:

$$\pi_2(t) = \hat{\pi}_2(t) + \frac{1}{\hat{\pi}_1(t)} \tag{20}$$

The first-level prediction $\hat{\mu}_1$ is defined by a logistic sigmoid transform of the second-level prediction μ_2 as follows:

$$\hat{\mu}_1(t) = s(\mu_2(t-1)) \tag{21}$$

The difference between the first-level prediction $\hat{\mu}_1(t)$ and first-level posterior $\mu_1(t)$ yields a prediction error $\delta_1(t)$ as follows:

$$\delta_1(t) = \mu_1(t) - \hat{\mu}_1(t) \tag{22}$$

Crucially, $\delta_1(t)$ is combined with the second-level precision π_2, yielding the precision-weighted "mid-level" prediction error $\varepsilon_2(t)$, which updates second-level prediction $\hat{\mu}_2(t)$ as follows:

$$\varepsilon_2(t) = \frac{1}{\pi_2} * \delta_1(t) \tag{23}$$

The precision of the prediction on the first and second levels evolve according to the following:

$$\hat{\pi}_1(t) = \frac{1}{\hat{\mu}_1(t) * (1 - \hat{\mu}_1(t))} \tag{24}$$

$$\hat{\pi}_2(t) = \frac{1}{\sigma_2(t) + \exp(\kappa_2 * \mu_3(t-1) + \omega_2)} \tag{25}$$

The volatility prediction error $\delta_2(t)$ governs the update to the third level of the HGF and is given by the following:

$$\delta_2(t) = \left(\frac{1}{\pi_2(t)} + \ (\mu_2(t) - \hat{\mu}_2(t))^2\right) * \hat{\pi}_2(t) - 1 \tag{26}$$

The third-level prediction $\hat{\mu}_3(t)$ and its precision $\hat{\pi}_3(t)$ are defined by the following:

$$\hat{\mu}_3(t) = \mu_3(t-1) \tag{27}$$

$$\hat{\pi}_3(t) = \frac{1}{\sigma_3(t-1) + \omega_3} \tag{28}$$

Finally, the third-level posterior $\mu_3(t)$ and its precision $\pi_3(t)$ are given by the following:

$$w_2 = \ \hat{\pi}_2(t) * \exp(\kappa_2 * \mu_3(t-1) * \omega_2) \tag{29}$$

$$\pi_3(t) = \hat{\pi}_3(t) + 0.5 * \kappa_2^2 * w_2(t) * (w_2(t) + (2 * w_2(t) - 1) * \delta_2(t)) \tag{30}$$

The model prediction $\hat{y}_{prediction}$ on the participants' predicted tilting direction of the upcoming visual stimulus is given by applying a unit sigmoid function with inverse decision temperature $\zeta = 1$ to $\hat{\mu}_1$ as follows:

$$\hat{y}_{prediction} = \frac{\hat{\mu}_1^{\zeta}}{\hat{\mu}_1^{\zeta} + (1 - \hat{\mu}_1)^{\zeta}} \tag{31}$$

Finally, combining the two log-likelihoods of $\hat{y}_{prediction}$ and $\hat{y}_{perception}$ given the actual responses $y_{prediction}$ and $y_{perception}$ yields the modeling cost. From here, the precision of the prior distributions can be optimized via the minimization of free energy (which represents a lower bound on the log-likelihood) with regard to the predicted responses.

Supplementary Note S2: Model and parameter recoveries

Simulations were performed to assess the validity of our modeling approach, both in terms of discriminability between models and parameter recovery. More specifically, the behavioral responses of each of the 52 participants were simulated 100 times, using the parameter estimates obtained from the initial fit of the winning model (*Associative learning* model). Priors ranged from 0.18 to 2.88 for π_a, from -5.26 to 0.41 for ω_2 and from -6.66 to -5.94 for ω_3. For each participant, the simulated behavioral responses were fitted by eight models (i.e., none: 0, A, P, S, AP, AS, PS, APS). Model inversions were performed separately for each run.

First, using Bayesian Model Selection comparing the eight models on the simulated data, we observed that model the *Associative learning* model best explained the data in both groups (protected exceedance probabilities: 1.00) in all the simulations, which is in line with our findings reported in Figure 2. For each participant, the simulated parameters of the *Associative learning* model were averaged over the five runs. As expected, the simulated parameters were correlated positively with the estimated parameters (tested using Pearson correlations). Indeed, the estimated and simulated associative learning precision π_a parameters were significantly correlated in 100% of the simulations in the NT and ASD groups (mean r = .88 in NT, r = .87 in ASD). The estimated and simulated second and third-level learning rates were also significantly correlated in 100% of the simulations in the NT and ASD groups (mean r = .91 for ω_2 and r = .68 for ω_3 in NT, mean r = .95 for ω_2 and r = .77 for ω_3 in ASD).

Finally, we assessed whether the simulated parameters differed between groups, using two-sample t-tests in each of the 100 simulations. In line with our findings reported in the Results section, the simulated π_a, ω_2 and ω_3 did not differ significantly between groups. Indeed, for each of the simulated parameters, 0% of the simulations showed significant group differences (π_a: t values ranging from -0.42 to 1.88, ω_2: t values ranging from -0.18 to 1.49, ω_3: t values ranging from -1.64 to 1.20).

Supplementary Note S3: Activation by ambiguous trials in MT/V5

In order to determine if the presentation of ambiguous trials generated activity in MT/V5, we specified another GLM with *Unambiguous rotation* and *Ambiguous rotation* as regressors. These regressors were coded as events starting at the appearance of the two vertical dots. To account for additional variance, we also included the following regressors: *Tone* (starting at the beginning of the tone, and lasting for 500 ms), *Prediction response* (starting at the onset of the participant's prediction response, and coded as an event), *Perception response* (starting at the onset of the participant's perception response, and coded as an event). In addition, as in the other analyses, the potential confounds were modeled as separate regressors of the GLM matrix and consisted of the six motion parameters, the ART-based outliers (if any) and the 10 first principal components identified with aCompCor in the denoising step. At the first level, contrast images were computed for each regressor at the individual level using t-statistics. At the second level, the mean of the contrasts across participants was compared to zero using a one-sample Student's t-test. We used the Anatomy Toolbox (SPM12) to create a structural mask encompassing the left and right MT/V5 areas. At the group level, the contrast *Ambiguous rotation* vs. *baseline* were masked with this MT/V5 mask, and voxel-based level thresholding was set at $p < .001$, while cluster-level extent thresholding was set at $p < .05$.

We found significant activation in the MT/V5 mask when perceiving ambiguous trials in the NT group (left cluster: $x = -44$, $y = -70$, $z = 2$, T = 8.7, $p < .01$, $p_{FWE\text{-}corr} = .06$; right cluster: $x = 48$, $y = -66$, $z = -2$, T = 6.8, $p < .01$, $p_{FWE\text{-}corr} = .17$) and in the ASD group (left cluster: $x = -42$, $y = -68$, $z = 2$, T = 10.1, $p < .01$, $p_{FWE\text{-}corr} = .06$; right cluster: $x = 50$, $y = -68$, $z = 2$, T = 7.9, $p < .01$, $p_{FWE\text{-}corr} = .17$). Results are illustrated in Supplementary Figure S2.

Supplementary References

1. Weilnhammer, V. A., Stuke, H., Sterzer, P. & Schmack, K. The Neural Correlates of Hierarchical Predictions for Perceptual Decisions. *J. Neurosci. Off. J. Soc. Neurosci.* **38**, 5008–5021 (2018).
2. Schmack, K., Weilnhammer, V., Heinzle, J., Stephan, K. E. & Sterzer, P. Learning What to See in a Changing World. *Front. Hum. Neurosci.* **10**, (2016).
3. Mathys, C., Daunizeau, J., Friston, K. J. & Stephan, K. E. A bayesian foundation for individual learning under uncertainty. *Front. Hum. Neurosci.* **5**, 39 (2011).
