# Supplementary material for: Neural correlates of hierarchical predictive processes in autistic adults
Source: Nat Commun. 2023 Jun 19;14:3640. doi: 10.1038/s41467-023-38580-9 (PMC10279690; doi:10.1038/s41467-023-38580-9)
Supplement: Supplementary file 3 — Reporting Summary [file 41467_2023_38580_MOESM3_ESM.pdf]

nature portfolio

Corresponding author(s): Laurie-Anne Sapey-Triomphe

Last updated by author(s): Apr 26, 2023

Reporting Summary

Nature Portfolio wishes to improve the reproducibility of the work that we publish. This form provides structure for consistency and transparency in reporting. For further information on Nature Portfolio policies, see our Editorial Policies and the Editorial Policy Checklist.

Statistics

For all statistical analyses, confirm that the following items are present in the figure legend, table legend, main text, or Methods section.

n/a	Confirmed	
☐	☒	The exact sample size (*n*) for each experimental group/condition, given as a discrete number and unit of measurement
☐	☒	A statement on whether measurements were taken from distinct samples or whether the same sample was measured repeatedly
☐	☒	The statistical test(s) used AND whether they are one- or two-sided<br>*Only common tests should be described solely by name; describe more complex techniques in the Methods section.*
☐	☒	A description of all covariates tested
☐	☒	A description of any assumptions or corrections, such as tests of normality and adjustment for multiple comparisons
☐	☒	A full description of the statistical parameters including central tendency (e.g. means) or other basic estimates (e.g. regression coefficient) AND variation (e.g. standard deviation) or associated estimates of uncertainty (e.g. confidence intervals)
☐	☒	For null hypothesis testing, the test statistic (e.g. *F*, *t*, *r*) with confidence intervals, effect sizes, degrees of freedom and *P* value noted<br>*Give P values as exact values whenever suitable.*
☐	☒	For Bayesian analysis, information on the choice of priors and Markov chain Monte Carlo settings
☒	☐	For hierarchical and complex designs, identification of the appropriate level for tests and full reporting of outcomes
☐	☒	Estimates of effect sizes (e.g. Cohen's *d*, Pearson's *r*), indicating how they were calculated

Our web collection on statistics for biologists contains articles on many of the points above.

Software and code

Policy information about availability of computer code

Data collection	The softwares used to present the experimental paradigm and collect the behavioral data were Matlab (version 2019a) and Psychtoolbox (version 3). Our Matlab codes were deposited in Zenodo (https://doi.org/10.5281/zenodo.7808070).<br>The MRI data were acquired on a 32 head coil 3T Philips Achieva system at the University Hospital of Leuven.
Data analysis	Statistical analyses were performed using R (version 4.0.3, http://www.r-project.org/).<br>The computational models were implemented on the implemented in the HGF 4.0 toolbox (TAPAS toolbox – Translational Algorithm for Psychiatry-Advancing Science, translationalneuromodeling.org/tapas/) in Matlab (R2020b version). Bayesian model selections were conducted using SPM12 (http://www.fil.ion.ucl.ac.uk/spm/) in Matlab (R2020b version).<br>The fMRI data were preprocessed and analyzed using Matlab (R2020b version) and Statistical Parametric Mapping (SPM), version 12. We also conducted a denoising step using the CONN functional connectivity toolbox v17 (http://www.nitrc.org/projects/conn). The preprocessing step included the use of the Artifact detection toolbox (ART: art-2015-10 release, https://www.nitrc.org/projects/artifact_detect/). To extract the contrast estimates of clusters and to create the localizer masks, we used Marsbar (release 0.44). The dynamic causal modeling analyses were conducted using Matlab (R2020b version) and Statistical Parametric Mapping (SPM), version 12. The SPM toolbox bspmview (version 20161108) was used to illustrate the brain activation patterns in Figures 3, 4 and S1.

For manuscripts utilizing custom algorithms or software that are central to the research but not yet described in published literature, software must be made available to editors and reviewers. We strongly encourage code deposition in a community repository (e.g. GitHub). See the Nature Portfolio guidelines for submitting code & software for further information.

Data

Policy information about availability of data

All manuscripts must include a data availability statement. This statement should provide the following information, where applicable:

- Accession codes, unique identifiers, or web links for publicly available datasets
- A description of any restrictions on data availability
- For clinical datasets or third party data, please ensure that the statement adheres to our policy

Source data are provided with this paper. The behavioral data generated in this study have been deposited in the Zenodo database under accession code (https://doi.org/10.5281/zenodo.7808070). The raw fMRI data are protected and are not available due to data privacy laws, but group-level fMRI results are available by request to the corresponding author.

Human research participants

Policy information about studies involving human research participants and Sex and Gender in Research.

Reporting on sex and gender	The sex of the participants was determined based on self-reporting. Source data disaggregates sex data. There were 13 men and 13 women in each group, so the sex-ratio was balanced and matched between groups. No sex-based analyses were performed because we had no prior hypotheses suggesting that results might differ based on the participant's sex, and because the sample size was too small to make comparisons between men and women.
Population characteristics	See above.
Recruitment	Adults with ASD were recruited via this expertise centre and via the LAuRes (Leuven Autism Research) consortium website. NT participants were recruited via the University of Leuven or acquaintances. Individuals who had contra-indication for MRI, or a total intelligence quotient below 70 at the Wechsler Adult Intelligence Scale IV could not participate this study, as these were exclusion criteria. A potential bias might be that some individuals may not have signed in to participate in this study due to their fear of coming to the hospital during the covid-19 pandemic. There might have been a self-selecting bias of the NT participants in joining a study on ASD.
Ethics oversight	The study was approved by the medical Research Ethical Committee UZ / KU Leuven.

Note that full information on the approval of the study protocol must also be provided in the manuscript.

Field-specific reporting

Please select the one below that is the best fit for your research. If you are not sure, read the appropriate sections before making your selection.

☐ Life sciences ☒ Behavioural & social sciences ☐ Ecological, evolutionary & environmental sciences

For a reference copy of the document with all sections, see nature.com/documents/nr-reporting-summary-flat.pdf

Behavioural & social sciences study design

All studies must disclose on these points even when the disclosure is negative.

Study description	This is a quantitative study, involving behavioral data from an associative learning task, questionnaire data (Autism-Spectrum Quotient, Intolerance of Uncertainty scale, Glasgow Sensory Questionnaire), and functional MRI data that were acquired during the associative learning task.
Research sample	Participants were 26 neurotypical adults (mean age: 31 years old, 13 men and 13 women) and 26 autistic adults (mean age: 32 years old, 13 men and 13 women). Adults with ASD were recruited via this expertise centre and via the LAuRes (Leuven Autism Research) consortium website (Flanders, Belgium). NT participants were recruited via the University of Leuven (Belgium) or acquaintances. The two groups were matched on age, sex ration and total intelligence quotient. Note that the autistic group is not representative of the whole spectrum of autism as we only included participants without intellectual deficiency and who could perform a behavioral task in the MRI.
Sampling strategy	The sampling procedure of the ASD participants was random, based on the individuals who received a diagnosis from the Expertise Center for Autism (University hospitals of Leuven) and who volunteered to participate in this study. We also aimed at having as many women as men. The sampling procedure of the NT participants was pseudo-random, so that the characteristics of the NT group would match the ones of the ASD group. The sample size was defined based on the study by Weilnhammer et al. 2018 who included 25 NT participants (but N = 23 in their analyses) to perform the same task in the MRI.
Data collection	The material involved for the data collection was a laptop for participants to train on the task before being installed in the MR scanner and a 32 head coil 3T Philips Achieva system to acquire the MRI data. During the fMRI acquisition, stimuli were projected on

a screen behind the scanner, and reflected through a mirror mounted on the head coil. Auditory stimuli were presented binaurally via headphones.
As the experimental conditions were the same for all the participants, the researcher was not blind to the experimental condition. There was not anyone else besides the participant and two researchers during the data acquisition.

Timing

The data were acquired from the 30/01/2020 to the 13/03/2020 (13 participants) and then from the 26/06/2020 to the 23/10/2020 (39 participants). The gap of 3 months between the two acquisition period was due to the closure of MRI facilities during the covid-19 pandemic.

Data exclusions

One ASD participant had two (out of five) runs excluded from the fMRI analyses, as there were more than 15% of outlier scans due to excessive head motion during the fMRI data acquisition.

Non-participation

No participant dropped out.

Randomization

Participants were allocated either to the neurotypical group (NT) if they did not report any neurological or psychiatric disorders, or to the autism group if they had received a diagnosis of Autism Spectrum Disorders.

Reporting for specific materials, systems and methods

We require information from authors about some types of materials, experimental systems and methods used in many studies. Here, indicate whether each material, system or method listed is relevant to your study. If you are not sure if a list item applies to your research, read the appropriate section before selecting a response.

Materials & experimental systems

n/a	Involved in the study
☒	☐ Antibodies
☒	☐ Eukaryotic cell lines
☒	☐ Palaeontology and archaeology
☒	☐ Animals and other organisms
☒	☐ Clinical data
☒	☐ Dual use research of concern

Methods

n/a	Involved in the study
☒	☐ ChIP-seq
☒	☐ Flow cytometry
☒	☐ MRI-based neuroimaging
